# Association between grip strength and cognitive impairment in older American adults

**DOI:** 10.3389/fnmol.2022.973700

**Published:** 2022-11-30

**Authors:** Jian Huang, Xinping Wang, Hao Zhu, Dong Huang, Weiwang Li, Jing Wang, Zhirong Liu

**Affiliations:** ^1^Department of Neurology, Xijing Hospital, Airforce Military Medical University, Xi'an, China; ^2^Department of Geriatrics, Air Force 986 Hospital, Xi'an, China; ^3^Department of Neurology, Xianyang First People's Hospital, Xianyang, China; ^4^Department of Neurology, The Second People's Hospital of Shaanxi Province, Xi'an, China; ^5^Department of Neurology, Xi'an Daxing Hospital, Xi'an, China; ^6^Department of Neurology, Xi'an First Hospital, Xi'an, China

**Keywords:** grip strength, cognitive impairment, Consortium to Establish a Registry for Alzheimer's Disease (CERAD), animal fluency (AF), digit symbol substitution test (DSST), smooth curve fit, machine learning

## Abstract

**Background and aims:**

Exponential population aging has led to an increased prevalence of cognitive impairment worldwide. Hand grip strength, which may be associated with physical activity, could be a useful predictor of cognitive impairment. However, few studies have reported the association, if any, between hand grip strength and cognitive function.

**Methods:**

We used data obtained from the National Health and Nutrition Examination Survey between 2011–2012 and 2013–2014 to investigate the association between hand grip strength and cognitive impairment. Cognitive impairment was assessed using the Consortium to Establish a Registry for Alzheimer's Disease (CERAD), animal fluency (AF), and digit symbol substitution test (DSST) scores. Cutoff values of CERAD < 5, AF < 14, and DSST < 34 were used to define cognitive impairment. In this cross-sectional study, we used odds ratios to determine the potential usefulness of hand grip strength for the prediction of cognitive impairment.

**Results:**

This study included 2,623 participants aged ≥60 years. The DSST results showed that hand grip strength was associated with a low risk of cognitive impairment and that subgroup analysis showed that male sex, 60–69 years of age, and the Non-Hispanic (NH)-White, NH Black, and Asian were associated with a significantly low risk of cognitive impairment. The CERAD test results showed that 70–79 years of age and the NH White were significantly associated with a low risk of cognitive impairment. By following full adjustment, we did not observe statistically significant differences between hand grip strength and cognitive impairment based on the CERAD test. The AF test results showed that >80 years of age, female sex, and the NH White were associated with a significantly low risk of cognitive impairment. The most important finding is that a linear association lies between grip strength and cognitive impairment, as well as a sex-based linear association. Machine learning of the XGBoost model suggests that grip strength is one of the top two most important negative predictor variables.

**Conclusion:**

We observed an inverse relationship between hand grip strength and cognitive impairment, which might suggest a shared underlying mechanism that needs to be further investigated using a large-scale prospective clinical trial to validate our findings.

## Introduction

Neurological disorders are the leading cause of disability and the second leading cause of death worldwide. Cognitive impairment is prevalent worldwide, particularly in those aged ≥60 years (Feigin et al., 2020). Increases are expected globally as a result of population growth and aging, and cognitive decline warrants urgent attention from policymakers and governments (Carroll, 2019). Reportedly, 6.2 million Americans aged ≥65 years are diagnosed with Alzheimer's disease (AD), and 13.8 million Americans are expected to have AD by 2060 (Warren, 2022). Therefore, AD and cognitive decline are associated with a high socioeconomic burden in the United States (US), and the burden is expected to increase 2-fold to 3.3% of the population by 2060 (Matthews et al., 2019). Unfortunately, currently, early diagnosis and effective treatment of cognitive impairment are relatively limited (Piersol et al., 2018). Therefore, researchers and policymakers consider the prevention of cognitive impairment a more viable strategy (Liss et al., 2021).

Several studies have focused on the role of diet and physical activity (Frith and Loprinzi, 2017, 2018), as well as protective and risk factors in the prevention of cognitive impairment (Li et al., 2019; Dong et al., 2020b; Casagrande et al., 2021; Kim et al., 2021; Pereira et al., 2021). In view of the fact that hand grip strength may associate with physical activity, hand grip strength may also associate with cognitive function (Bohannon, 2019). Previous studies have reported a likely association between hand grip strength and cognitive abilities (Sternäng et al., 2016). However, another study observed no association between hand grip strength and cognitive function (Ritchie et al., 2016). A recent study that included elderly cancer survivors observed an association between hand grip strength and cognitive function in this patient population (Yang et al., 2018).

However, clinical findings that describe the association between hand grip strength and cognitive impairment remain inconsistent. Therefore, we extracted data from the National Health and Nutrition Examination Survey (NHANES) (2011–2012 and 2013–2014) to investigate the association between hand grip strength and cognitive impairment in elderly adults in the US. To the best of our knowledge, this is the first study that confirms the association between grip strength and cognitive impairment based on representative national data.

## Materials and methods

### Study population

**Figure 1:**
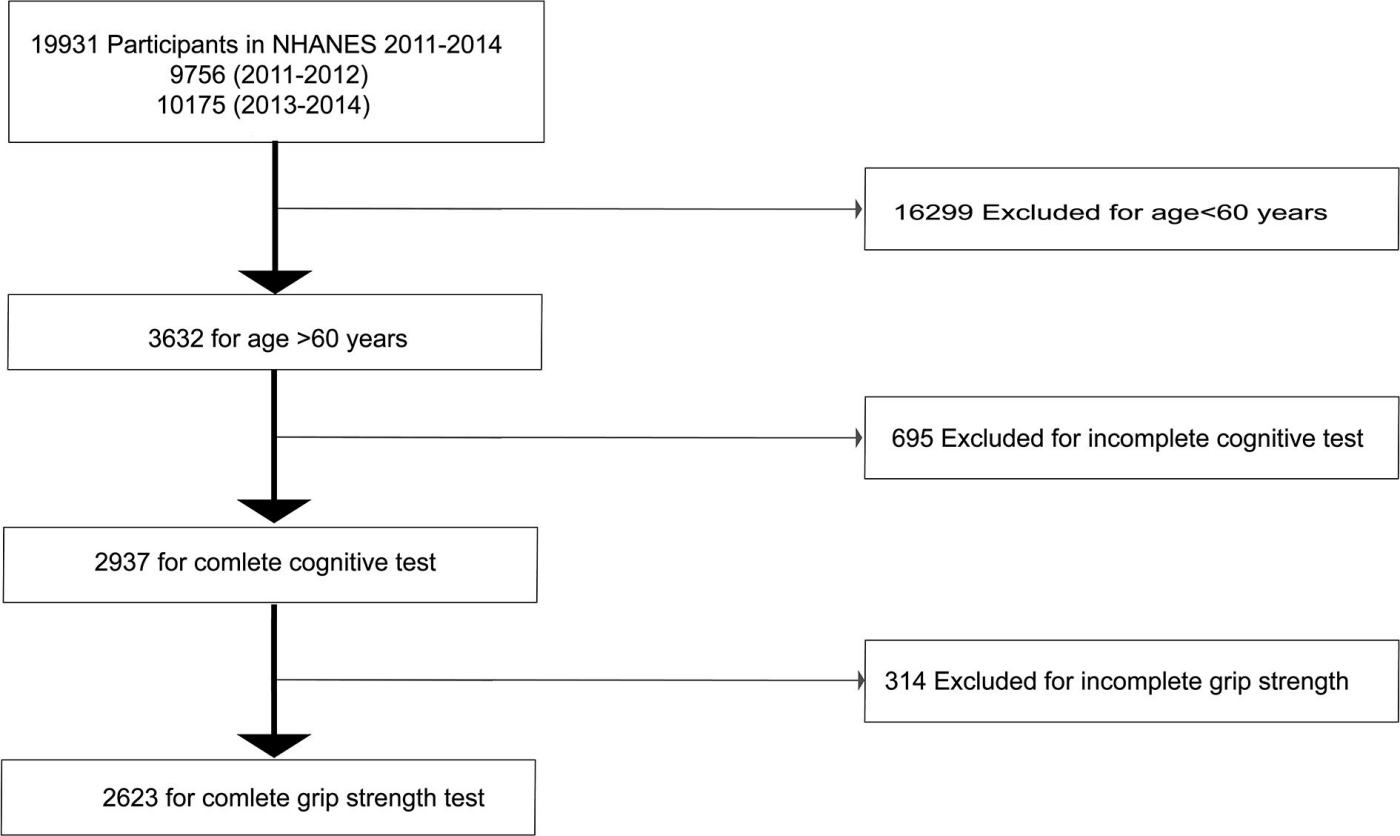
Flow chart showing the selection of study participants.

We extracted data from the NHANES across two phases (2011–2012 and 2013–2014). The NHANES included cross-sectional nationally representative health examination surveys that are used to assess the health and nutritional status of the population in the US. The purpose of the information collected by NHANES was for health statistics. Several studies based on the NHANES data have been published in recent years (Flegal et al., 2005; Wong et al., 2019; Appiah et al., 2021). A total of 19,931 participants completed the survey in 2011–2012 (*n* = 9,756) and 2013–2014 (*n* = 10,175). Exclusion criteria were as follows: (a) <60 years of age (16,299 individuals), (b) inability to complete cognitive function tests (*n* = 695), and (c) inability to perform hand grip strength experiments (*n* = 314). Eventually, data from 2,623 participants were analyzed (Figure 1). Written informed consent was obtained from all participants and by the Research Ethics Review Board of the National Centre for Health Statistics. Informed consent was not required for the second phase of the study for analysis of the public database. This study is reported based on the Strengthening the Reporting of Observational Studies in Epidemiology (STROBE) guidelines (Vandenbroucke et al., 2007).

### Primary exposure

We obtained a detailed protocol for the hand grip strength test from the NHANES Muscle Strength Procedures Manual (Yang et al., 2018). Briefly, a dynamometer was used for bilateral hand assessment. Participants were randomly assigned to grasp the dynamometer using the maximal strength possible, and each hand was tested three times. Participants who could not correctly grasp the dynamometer were excluded from the study, and we measured the combined grip strength (kg) (the sum of the largest reading from each hand).

### Outcome variables

Cognitive function evaluation was performed at a mobile examination center. A series of tests were performed to assess participants' working memory, delayed recall, and verbal fluency. Participants consented to audio-record the testing throughout the assessment for quality purposes, and the score was approved by all participants.

The Consortium to Establish a Registry for Alzheimer's Disease (CERAD) test battery was used for the assessment of new learning, recognition memory, and delayed recall. Three consecutive learning trials were included in the CERAD-Word Learning test (Morris et al., 1989). After participants read 10 unrelated words aloud, they were required to recall the words. The sum of the three trial scores was 30. During the CERAD-Delayed Recall test (Sotaniemi et al., 2012; Dong et al., 2020a), participants were instructed to recall 10 words in the CERAD-Word Learning test after completing the other trials.

The animal fluency (AF) test, which measures participants' verbal fluency, involves naming as many animals as the participant can in 1 min (Prince et al., 2003). The digit symbol substitution test (DSST) was used to assess working memory, processing speed, and sustained attention. Participants were instructed to copy symbols in 133 boxes after 2 min. The correct matches were included in the total score (Brody et al., 2019).

Unfortunately, cutoffs for cognitive tests that can be used as a gold standard remain unavailable. Based on the historical literature, the 25th percentile of the scores is widely accepted as the cutoff value (Bailey et al., 2020). In the present study, the 25th percentile of the scores was CERAD < 5, AF < 14, and DSST < 34, respectively. Combined CERAD, AF, and DSST tests scores, a score of less than the 25th percentile of the scores on at least one of the CERAD, AF, and DSST tests, is considered as cognitive impairment.

### Covariates

We extracted potential confounders, including sociodemographic factors, lifestyle, and health status from the previous literature.

Sociodemographic factors included sex, age, race, educational level, marital status, and the poverty-income ratio. The following age categories were used: 60–69, 70–79, and ≥80 years. Race was categorized into the following groups: Mexican American/other Hispanic, Non-Hispanic (NH)-White, NH-Black, NH-Asian, and other races. Educational level was classified as less than high school, high school, and above high school. The poverty–income ratio was calculated by dividing family (or individual) income by the poverty guidelines specific to the survey year and was categorized into <1 and >1. Marital status was classified into married/living with a partner, widowed/divorced/separated, and never married.

Lifestyle patterns were categorized based on personal habits, including alcohol consumption (12 alcoholic drinks/year), smoking (at least 100 cigarettes), and vigorous work activity (yes or no) (Fan et al., 2021). Health status was evaluated based on history of coronary heart disease, stroke, diabetes, hypertension, and high serum cholesterol levels. The body mass index (BMI) was classified into the following groups: <25, 25–30, and >30 kg/m^2^. Depression was assessed using the 9-question Patient Health Questionnaire. Based on the reports in the literature, scores ≥10 were defined as depression (Ge et al., 2020). Arm length, arm circumference, and waist circumference, which may be potential confounders of hand grip strength, were considered in the analysis.

### Statistical analysis

Continuous variables are expressed as weighted mean ± standard deviation and were compared using an independent *t*-test. Categorical variables are expressed as weighted percentages and were compared using the chi-square test. A multivariate logistic regression model was established to determine the association between hand grip strength and cognitive impairment. The model was adjusted for sex, age, race, educational level, marital status, poverty-income ratio, alcohol consumption, smoking, vigorous work activity, BMI, depression, and a history of coronary heart disease, stroke, diabetes, hypertension, and high serum cholesterol levels. Furthermore, we performed subgroup analysis based on age, sex, and race. Finally, we constructed the XGBoost algorithm model to predict the relative importance of selected variables (grip strength, arm circumference, waist circumference, and arm length). We performed the XGBoost model to analyze the contribution of each variable to cognitive impairment.

All statistical analyses were performed using the R software (http://www.R-project.org) and Empowerstats (http://www.empowerstats.com, X&Y Solutions, Inc., USA). A full sample 4-year mobile examination center assessment weight was used to represent the survey among older adults. A two-sided test was used, and *p* < 0.05 was considered statistically significant.

## Results

### Characteristics of study participants

We analyzed the records of 2,623 participants aged ≥60 years in this study. Table 1 shows the overall characteristics of the study samples represented as quartiles of grip strength. We observed CERAD scores <5 in 20.61%, AF scores <14 in 20.33%, and DSST scores <34 in 13.61% of participants. In addition to a history of diabetes and high serum cholesterol levels, statistically significant differences were observed in most participants through quartiles of hand grip strength (*p* > 0.05). Furthermore, high hand grip strength was associated with male sex, 60–69 years of age, NH White race, married status/living with a partner, poverty-income ratio >1, higher educational levels, vigorous work activity, BMI > 30 kg/m^2^, alcohol consumption, smoking (at least 100 cigarettes), and a history of coronary heart disease. In contrast, low hand grip strength was associated with female sex, Asian race, never married status, less vigorous work activity, poverty-income ratio <1, BMI 25–30 kg/m^2^, a history of stroke, diabetes, hypertension, high serum cholesterol levels, and depression. Notably, arm length, arm circumference, and waist circumference were associated with hand grip strength.

**Table 1:** General characteristics of participants (*n* = 2,623) stratified by grip strength (1–4, kg) in the NAHENS 2011–2014.

**Characters**	**Total** **(*n* = 2,263)**	**Quartiles 1 (< 46.7)** **(*n* = 653)**	**Quartiles 2 (46.7–57.7) (*n* = 658)**	**Quartiles 3 (57.7–75.2) (*n* = 654)**	**Quartiles 4 (>75.2)** **(*n* = 658)**	***P-*value**
Gender						< 0.00
Male	46.11	5.49	14.52	61.58	99.12	
Female	53.89	94.51	85.48	38.42	0.88	
Age (years)						< 0.00
60–69	56.67	34.17	59.56	59.03	71.76	
70–79	29.71	35	29.95	30.04	24.53	
≥80	13.61	30.83	10.49	10.94	3.71	
Race						0.00
Mexican American/other Hispanic	6.62	7.83	7.51	6.95	4.4	
Non-Hispanic (NH) white	80.54	81.32	80.04	77.38	83.06	
NH black	8.13	6.36	7.34	10.09	8.75	
Asian	2.95	3.67	3.53	3.35	1.41	
Other race	1.77	0.81	1.58	2.24	2.38	
Education						< 0.00
Less than high school	15.36	21.26	13.01	15.07	12.67	
High school	22.24	26.49	22.43	20.69	19.63	
Above high school	62.38	52.25	64.52	64.21	67.7	
Not recorded	0.02		0.04	0.03		
Marital status						< 0.00
Married/living with partner	65.4	49.91	58.61	67.36	83.94	
Widowed/divorced/separated	30.3	46.33	36.87	26.63	12.95	
Never married	4.28	3.76	4.47	5.95	3.11	
Not recorded	0.03		0.05	0.05		
Poverty-income ratio						< 0.00
< 1	7.96	12.49	7.49	7.56	4.75	
>1	85.74	80.38	85.42	85.69	90.83	
Not recorded	6.3	7.14	7.09	6.75	4.41	
Alcohol (12 alcohol drinks per year)						< 0.00
Yes	73.81	56.9	71.8	79.13	86.03	
Smoked (at least 100 cigarettes)						< 0.00
Yes	50.78	41.05	46.29	51.6	62.98	
Vigorous work activity						< 0.00
Yes	13.35	4.25	10.37	12.27	25.2	
BMI (kg/m^2^)						< 0.00
< 25	26.27	35.46	26.86	24.35	19.25	
25–30	35.47	29.47	35.01	37.8	39.2	
>30	37.24	32.67	37.28	37.32	41.16	
Not recorded	1.02	2.39	0.85	0.53	0.4	
History of coronary heart disease						0.00
Yes	9.55	8.28	7.46	11.67	10.86	
History of stroke						0.00
Yes	6.01	9.65	4.86	5.27	4.52	
History of diabetes						0.54
Yes	19.11	21.82	18.78	17.94	18.03	
History of hypertension						< 0.00
Yes	58.31	67.47	55.63	54.73	55.86	
History of high cholesterol						0.53
Yes	58.19	59.96	57.73	57	58.08	
CERAD (< 5)	20.61	26.65	16.15	23.18	17.35	< 0.00
AF (< 14)	20.33	31.04	17.73	17.79	15.56	< 0.00
DSST (< 34)	13.61	24.52	10.95	12.07	7.87	< 0.00
Depressive (>10)	6.39	9.85	6.9	3.6	5.25	< 0.00
Arm length (cm)	37.52 ± 2.77	35.62 ± 2.20	36.30 ± 2.20	37.98 ± 2.23	39.87 ± 2.23	< 0.00
Arm circumference (cm)	32.60 ± 4.68	30.62 ± 5.07	32.18 ± 4.77	32.74 ± 4.13	34.52 ± 3.85	< 0.00
Waist circumference (cm)	102.50 ± 14.68	96.67 ± 13.70	100.39 ± 14.00	104.11 ± 13.64	107.88 ± 14.79	< 0.00

Mean ± SD for arm length, arm circumference, and waist circumference; *p*-value was calculated using the weighted linear regression model.

% for gender; age; race; education; marital status; poverty-income ratio; alcohol; smoked; vigorous work activity; BMI (kg/m^2^); history of coronary heart disease; history of stroke; history of diabetes; history of hypertension; history of high cholesterol; CERAD; AF; DSST; and depressive. *p*-value was calculated using the weighted chi-square test.

### Association between hand grip strength and cognitive impairment based on the Consortium to Establish a Registry for Alzheimer's Disease test results

Table 2 shows the association between hand grip strength and cognitive impairment measured using the CERAD test. The non-adjusted model showed an odds ratio (OR) of 0.99 [95% confidence interval (CI) 0.99–1.00]. After adjustment for age, sex, and race, a statistically significant difference was observed between hand grip strength and a low risk of cognitive impairment (OR = 0.98, 95% CI 0.97–0.99) and CERAD in Q2–Q4 (Q2: OR = 0.63, 95% CI 0.47–0.85; Q3: OR = 0.68, 95% CI 0.49–0.95; Q4: OR = 0.52, 95% CI 0.35–0.77). However, following full adjustment, we did not observe statistically significant differences between hand grip strength and cognitive impairment.

**Table 2:** Associations between grip strength (kg) and cognitive impairment (CERAD<5) (*n* = 2,623), NHANES 2011–2014.

	**Model 1**		**Model 2**		**Model 3**
	**OR (95% CI), *P***		**OR (95% CI), *P***		**OR (95% CI), *P***
Grip strength (kg)	0.99 (0.99, 1.00), 0.12		0.98 (0.97, 0.99), 0.00		0.99 (0.98, 1.00), 0.09
**Quartiles of grip strength**
Q1 (< 46.7)	1		1		1
Q2 (46.7–57.7)	0.61 (0.47, 0.79), 0.00		0.63 (0.47, 0.85), 0.00		0.86 (0.62, 1.18), 0.36
Q3 (57.7–75.2)	0.97 (0.76, 1.24), 0.83		0.68 (0.49, 0.95), 0.02		0.94 (0.61, 1.34), 0.77
Q4 (>75.2)	0.80 (0.62, 1.03), 0.08		0.52 (0.35, 0.77), 0.00		0.80 (0.51, 1.26), 0.34
*P* trend	0.92		0.00		0.15
**Stratified by gender**
Male	0.97 (0.97, 0.98), < 0.00		0.98 (0.98, 0.99), 0.00		0.99 (0.98, 1.00), 0.46
Female	0.95 (0.93, 0.96), < 0.00		0.97 (0.95, 0.98), 0.00		0.98 (0.96, 1.00), 0.12
**Stratified by age**
60–69	1.00 (1.00, 1.01), 0.01		0.98 (0.97, 1.00), 0.04		0.99 (0.98, 1.00), 0.33
70–79	1.00 (0.99, 1.01), 0.31		0.97 (0.96, 0.99), 0.00		0.98 (0.96, 0.99), 0.02
≥80	1.00 (0.99, 1.01), 0.37		0.98 (0.96, 1.00), 0.09		1.00 (0.97, 1.02), 0.91
**Stratified by race**
Mexican American/other Hispanic	1.00 (0.99, 1.02), 0.12		0.99 (0.98, 1.01), 0.74		1.01 (0.99, 1.04), 0.15
NH White	0.98 (0.98, 0.99), 0.00		0.97 (0.96, 0.98), < 0.00		0.97 (0.96, 0.99), 0.00
NH Black	1.00 (0.99, 1.01), 0.36		0.99 (0.98, 1.00), 0.42		1.00 (0.99, 1.02), 0.53
Asian	0.99 (0.96, 1.01), 0.47		0.96 (0.93, 1.01), 0.13		1.02 (0.95, 1.09), 0.53
Other RACE	0.97 (0.92, 1.01), 0.23		0.96 (0.89, 1.03), 0.34		0.19 (0.00, Inf), 0.99

Model 1, non-adjusted model; Model 2, adjusted for age, race, and gender; Model 3, adjusted for age, race, gender, education, marital status, poverty-income ratio, alcohol, smoked, vigorous work activity, BMI, history of coronary heart disease, diabetes, stroke, hypertension, high cholesterol, depressive, arm length, arm circumference, and waist circumference.

Subgroup analysis revealed that 70–79 years of age (full adjustment: OR = 0.97, 95% CI 0.96–0.99) and NH White race (full adjustment: OR = 0.97, 95% CI 0.96–0.98) were associated with a significantly low risk of cognitive impairment. However, there were no statistically significant differences in sex after full adjustment.

### Association between hand grip strength and cognitive impairment based on the AF test results

Table 3 shows the association between hand grip strength and cognitive impairment based on the AF test results. The non-adjusted model showed an OR of 0.98 (95% CI 0.98–0.99). After adjustment for age, sex, and race, a statistically significant difference was observed between hand grip strength and a low risk of cognitive impairment (OR 0.98, 95% CI 0.97–0.99) and AF in Q2–Q4 (Q2: OR = 0.67, 95% CI 0.51–0.86; Q3: OR = 0.63, 95% CI 0.47–0.86; Q4: OR = 0.53, 95% CI 0.36–0.76). However, following full adjustment, we did not observe statistically significant differences between hand grip strength and cognitive impairment.

**Table 3:** Associations between grip strength and cognitive impairment (AF<14) (*n* = 2,623), NHANES 2011–2014.

	**Model 1**		**Model 2**		**Model 3**
	**OR (95% CI), *P***		**OR (95% CI), *P***		**OR (95% CI), *P***
Grip strength (kg)	0.98 (0.98, 0.99), < 0.00		0.98 (0.97, 0.99), 0.00		0.99 (0.98, 1.00), 0.08
**Quartiles of grip strength**
Q1 (< 46.7)	1		1		1
Q2 (46.7–57.7)	0.64 (0.51, 0.82), 0.00		0.67 (0.51, 0.86), 0.00		0.82 (0.61, 1.09), 0.18
Q3 (57.7–75.2)	0.72 (0.57, 0.91), 0.00		0.63 (0.47, 0.86), 0.00		0.87 (0.62, 1.23), 0.45
Q4 (>75.2)	0.58 (0.45, 0.73), < 0.00		0.53 (0.36, 0.76), 0.00		0.77 (0.50, 1.18), 0.24
*P* trend	< 0.00		< 0.00		0.10
**Stratified by gender**
Male	0.98 (0.97, 0.99), < 0.00		0.98 (0.98, 0.99), 0.00		0.99 (0.98, 1.0), 0.52
Female	0.97 (0.95, 0.98), < 0.00		0.97 (0.95, 0.98), 0.00		0.98 (0.96, 0.99), 0.02
**Stratified by age**
60–69	0.99 (0.99, 1.00), 0.78		0.99 (0.98, 1.00), 0.07		0.99 (0.98, 1.01), 0.79
70–79	0.98 (0.97, 0.99), 0.00		0.98 (0.96, 0.99), 0.00		0.98 (0.96, 1.00), 0.05
≥80	0.98 (0.97, 0.99), 0.02		0.96 (0.95, 0.98), 0.00		0.97 (0.94, 0.99), 0.02
**Stratified by race**
Mexican American/other Hispanic	0.98 (0.97, 0.99), 0.00		0.98 (0.96, 1.00), 0.12		0.99 (0.97, 1.01), 0.70
NH White	0.97 (0.97, 0.98), < 0.00		0.96 (0.95, 0.98), < 0.00		0.98 (0.96, 0.99), 0.03
NH Black	0.99 (0.98, 0.99), 0.01		0.99 (0.98, 1.00), 0.21		0.99 (0.98, 1.00), 0.42
Asian	1.00 (0.98, 1.02), 0.58		1.02 (0.99, 1.053 0.12		1.01 (0.97, 1.05), 0.56
Other Race	0.97 (0.93, 1.01), 0.25		0.92 (0.85, 1.00), 0.06		0.81 (0.00, Inf), 0.99

Model 1, non-adjusted model; Model 2, adjusted for age, race, and gender; Model 3, adjusted for age, race, gender, education, marital status, poverty-income ratio, alcohol, smoked, vigorous work activity, BMI, history of coronary heart disease, diabetes, stroke, hypertension, high cholesterol, depressive, arm length, arm circumference, and waist circumference.

Subgroup analysis indicated that >80 years of age (full adjustment: OR = 0.97, 95% CI 0.94–0.99), female sex (OR = 0.98, 95% CI 0.96–0.99), and NH White race (full adjustment: OR = 0.98, 95% CI 0.96–0.99) were associated with a significantly low risk of cognitive impairment.

### Association between hand grip strength and cognitive impairment based on the DSST results

Table 4 shows the association between hand grip strength and cognitive impairment based on the DSST results. A statistically significant difference was observed between hand grip strength and a low risk of cognitive impairment in each model (full adjustment: OR = 0.97, 95% CI 0.96–0.98) and DSST in Q2–Q4 (full adjustment: Q2: OR = 0.68, 95% CI 0.48–0.97; Q3: OR = 0.57, 95% CI 0.37–0.87; Q4: OR = 0.34, 95% CI 0.20–0.58).

**Table 4:** Associations between grip strength and cognitive impairment (DSST<34) (*n* = 2,623), NHANES 2011–2014.

	**Model 1**		**Model 2**		**Model 3**
	**OR (95% CI), *P***		**OR (95% CI), *P***		**OR (95% CI), *P***
Grip strength (kg)	0.98 (0.98, 0.99), < 0.00		0.96 (0.95, 0.97), < 0.00		0.97 (0.96, 0.98), 0.00
**Quartiles of grip strength**
Q1 (< 46.7)	1		1		1
Q2 (46.7–57.7)	0.57 (0.44, 0.74), 0.00002		0.48 (0.35, 0.64), < 0.00		0.68 (0.48, 0.97), 0.03
Q3 (57.7–75.2)	0.78 (0.61, 0.99), 0.04		0.39 (0.28, 0.56), < 0.00		0.57 (0.37, 0.87), 0.00
Q4 (>75.2)	0.53 (0.41, 0.69), < 0.00		0.22 (0.14, 0.33), < 0.00		0.34 (0.20, 0.58), 0.00
*P* trend	0.00		< 0.00		0.00
**Stratified by gender**
Male	0.97 (0.96, 0.97), < 0.00		0.96 (0.95, 0.97), < 0.00		0.97 (0.96, 0.98), 0.00
Female	0.94 (0.93, 0.96), < 0.00		0.95 (0.94, 0.97), < 0.00		0.98 (0.95, 1.00), 0.08
**Stratified by age**
60–69	0.99 (0.99, 1.00), 0.48		0.96 (0.95, 0.97), < 0.00		0.97 (0.96, 0.99), 0.00
70–79	0.99 (0.98, 1.00), 0.08		0.96 (0.95, 0.98), < 0.00		0.98 (0.96, 1.00), 0.13
≥80	0.98 (0.97, 1.00), 0.04		0.96 (0.94, 0.98), 0.00		0.97 (0.94, 1.00), 0.04
**Stratified by race**
Mexican American/other Hispanic	0.99 (0.98, 1.00), 0.17		0.98 (0.96, 0.99), 0.03		1.00 (0.98, 1.02), 0.74
NH White	0.97 (0.96, 0.97), < 0.00		0.94 (0.93, 0.96), < 0.00		0.96 (0.94, 0.98), 0.00
NH Black	0.99 (0.98, 1.00), 0.05		0.97 (0.96, 0.98), 0.00		0.97 (0.96, 0.99), 0.01
Asian	0.95 (0.92, 0.98), 0.00		0.92 (0.88, 0.96), 0.00		0.83 (0.72, 0.95), 0.01
Other Race	0.99 (0.95, 1.04), 0.82		0.96 (0.89, 1.04), 0.39		0.15 (0.00, Inf), 0.99

Model 1, non-adjusted model; Model 2, adjusted for age, race, and gender; Model 3, adjusted for age, race, gender, education, marital status, poverty-income ratio, alcohol, smoked, vigorous work activity, BMI, history of coronary heart disease, diabetes, stroke, hypertension, high cholesterol, depressive, arm length, arm circumference, and waist circumference.

Subgroup analysis revealed that 60–69 years of age (full adjustment: OR = 0.97, 95% CI 0.96–0.99), male sex (full adjustment: OR = 0.97, 95% CI 0.96–0.98), NH White race (full adjustment: OR = 0.96, 95% CI 0.94–0.98), NH Black race (full adjustment: OR = 0.97, 95% CI 0.96–0.99), and Asian race (full adjustment: OR = 0.83, 95% CI 0.72–0.95) were associated with a significantly low risk of cognitive impairment.

### Sensitivity analysis outcomes

**Figure 2:**
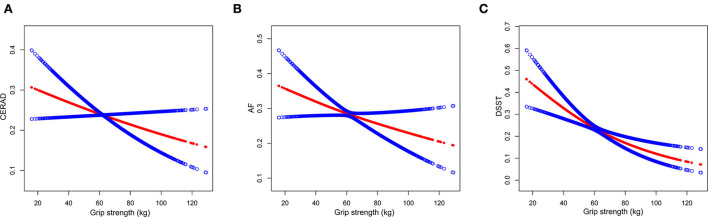
The association between grip strength and cognitive impairment. Consortium to Establish a Registry for Alzheimer's Disease (CERAD) **(A)**, animal fluency (AF) **(B)**, and digit symbol substitution test (DSST) **(C)** represent cognitive impairment. The red points show a smooth curve fitting line, the blue points show 95% confidence interval. The relationship was adjusted for age, race, gender, education, marital status, poverty-income ratio, alcohol, smoked, vigorous work activity, BMI, history of coronary heart disease, diabetes, stroke, hypertension, high cholesterol, depressive, arm length, arm circumference, and waist circumference.

**Figure 3:**
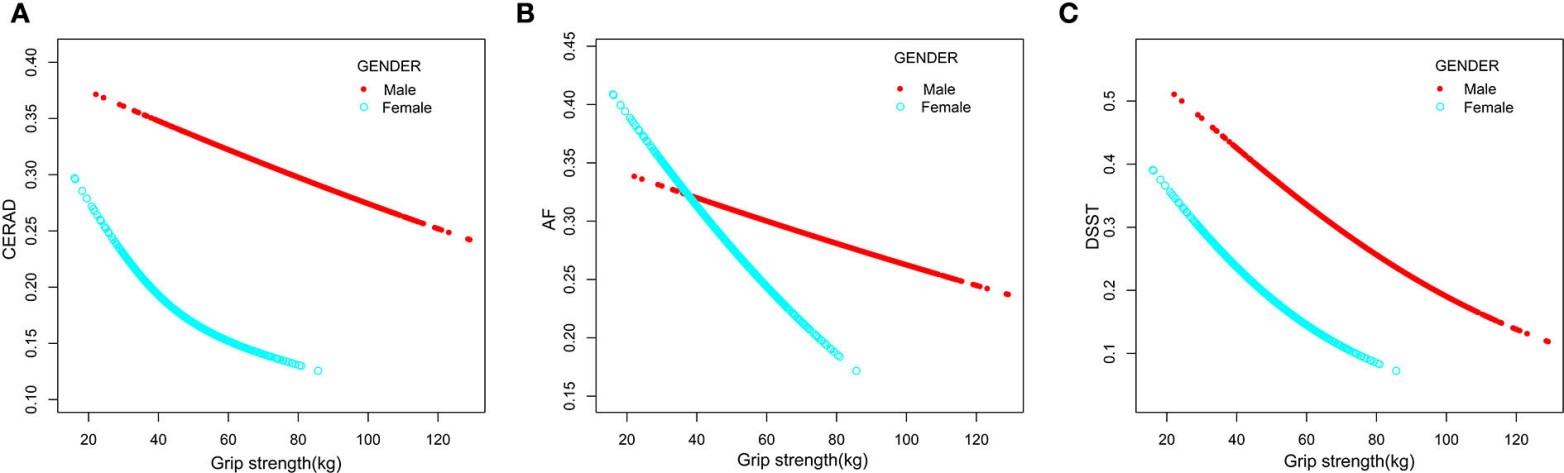
The association between grip strength and cognitive impairment for male sex (red line) and female sex (blue line). CERAD **(A)**, AF **(B)**, and DSST **(C)** represent cognitive impairment. The relationship was adjusted for age, race, gender, education, marital status, poverty-income ratio, alcohol, smoked, vigorous work activity, BMI, history of coronary heart disease, diabetes, stroke, hypertension, high cholesterol, depressive, arm length, arm circumference, and waist circumference.

We used a smooth curve fit model to investigate the possibility of a non-linear association between hand grip strength and cognitive impairment (Figure 2). After full adjustment for covariates (using the aforementioned full adjustment model), our analysis indicated a linear association between hand grip strength and cognitive impairment. Furthermore, the smooth curve fit model was applied to investigate a sex-stratified association between hand grip strength and cognitive impairment (Figure 3). These results were consistent with those obtained using the full adjustment model, which showed a linear association.

### Machine learning using the XGBoost algorithm model

**Figure 4:**
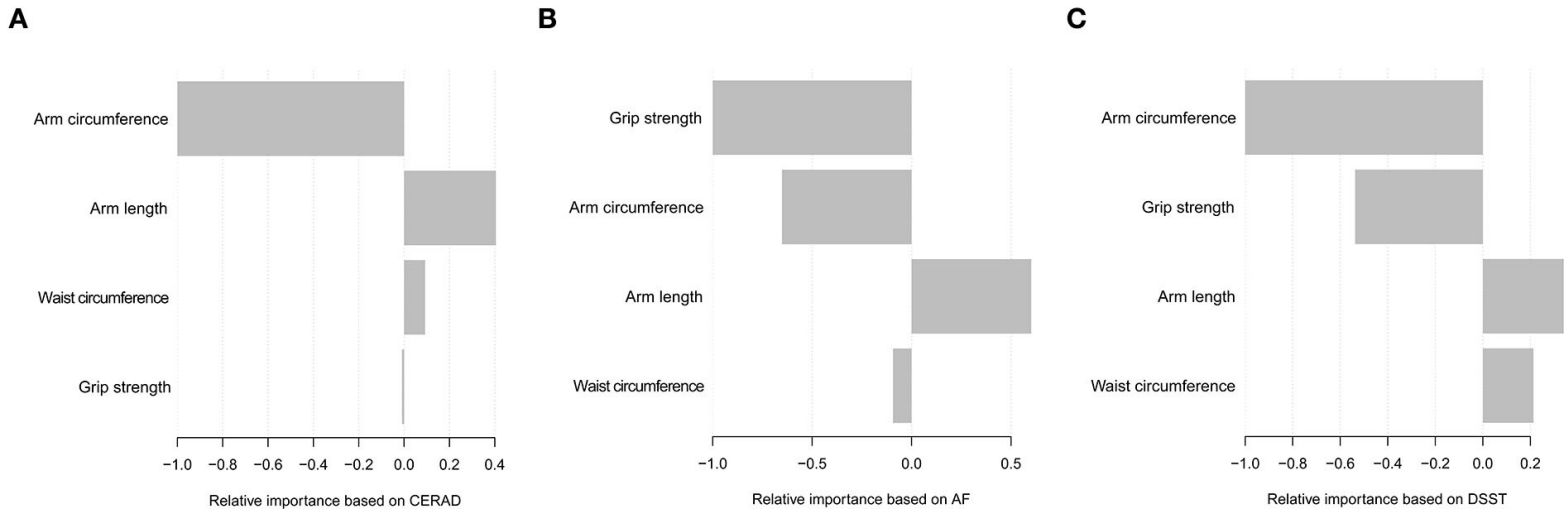
Relative importance of the selected variables using XGBoost and the corresponding variable importance score based on CERAD **(A)**, AF **(B)**, and DSST **(C)**. The *X*-axis indicates the importance score, which is the relative number of a variable that is used to distribute the data, and *Y*-axis indicates the selected variable.

We used the machine learning of the XGBoost model to determine the relative importance of variables associated with cognitive impairment. Variables included grip strength, arm circumference, waist circumference, and arm length. Data showed that each variable's contribution by the XGBoost model, arm circumference, and grip strength was the topmost important negative variables of the dataset based on AF and DSST. Arm length was the most important positive variable of the dataset based on cognitive function tests (Figure 4).

## Discussion

In the present study, we analyzed the data of 2,623 elderly American adults to investigate the association between grip strength and cognitive impairment. After adjustment for all potential confounders, our results showed an association between growth hand grip strength and cognitive impairment, and statistically significant differences were observed in NH White, NH Black, Asian, male sex, and individuals aged 60–69 years throughout subgroup analyses. Furthermore, a linear correlation was observed between hand grip strength and cognitive impairment. Finally, machine learning of the XGBoost model shows that grip strength is one of the top two most important negative predictor variables.

Several previous studies have investigated the association between hand grip strength and cognitive function. In 2000, Christensen et al. reported that grip strength could not predict future changes in memory or IQ (Christensen et al., 2000). Deary et al. (2011) also observed no association between grip strength and cognitive function. In 2016, Ritchie et al. (2016) reported no association between an increase or decrease in grip strength and cognitive impairment. Nevertheless, some researchers who used the horizontal linear mixed growth model have confirmed a clear association between cognitive impairment and a decline in grip strength (Macdonald et al., 2011). Another study indicated that grip strength did not predict cognitive impairment in individuals aged < 65 years; however, it could predict a decline in all cognitive domains in individuals aged >65 years (Sternäng et al., 2016). A study that included cancer survivors and used information from the NHANES database reported an association between cognitive impairment and hand grip strength (Yang et al., 2018). Several studies have investigated the association between cognitive functioning and hand grip strength (Firth et al., 2018; Robitaille et al., 2018; Yoon et al., 2018; Ahrenfeldt et al., 2019; Imaoka et al., 2019; Kim and Kim, 2019; Liu et al., 2019, 2020; Hooyman et al., 2021; Watermeyer et al., 2021). Our results were consistent with those reported by previous studies; we observed that cognitive impairment was associated with grip strength in older American adults.

High hand grip strength was associated with male sex, vigorous work activity, alcohol consumption, smoking, and a history of coronary heart disease. However, low hand grip strength was associated with female sex, never married status, a history of stroke, diabetes, hypertension, high serum cholesterol levels, and depression. The mechanism under the interesting results may involve the basal metabolic rate and blood flow to the upper extremities. Regular exercise and a healthy lifestyle are also important factors. Of course, the exact mechanism needs to be confirmed by more animal and clinical trials.

The following are the strengths of the present study. (a) The large sample size (2,623 participants) is a strength because an increase in sample size improves the statistical power of the study, and our results are statistically more significant. (b) In this study, we sorted different categories of missing data, which minimized the effect of missing data on bias in our results. (c) We eliminated as many confounders as possible, including a history of chronic disease and depressive status, and used the three most common tests performed in clinical practice for the assessment of cognitive function. (d) The linear association between grip strength and cognitive impairment was supported by the use of a smooth curve fit model and further confirmed using subgroup and sensitivity analyses.

The following are the weaknesses of the present study that may have affected our results. (a) Owing to the cross-sectional study design, it is difficult to distinguish causality between grip strength and cognitive impairment owing to its internal characteristics. (b) The study population was limited to Americans; therefore, our results may not be generalizable. (c) Cognitive function evaluation was based on the cognitive level of the interviewee; therefore, some cognitive functions that did not meet the interview requirements may have been excluded inadvertently.

In conclusion, our study highlights an inverse relationship between hand grip strength and cognitive impairment, which might suggest a shared underlying mechanism that needs to be further investigated using a large-scale prospective clinical trial to validate our findings.

## Data availability statement

Publicly available datasets were analyzed in this study. This data can be found here: Centers for Disease Control and Prevention (CDC) National Health and Nutrition Examination Survey (NHANES), https://wwwn.cdc.gov/nchs/nhanes/Default.aspx. Further inquiries can be directed to the corresponding author.

## Ethics statement

Ethical review and approval was not required for the study on human participants in accordance with the local legislation and institutional requirements. Written informed consent from the patients/participants or patients/participants' legal guardian/next of kin was not required to participate in this study in accordance with the national legislation and the institutional requirements.

## Author contributions

JH and ZL planned and executed the studies and data analysis and drafted the manuscript. XW processed and analyzed the data. HZ and DH made great efforts to revise the manuscript. WL conceived the item. JW facilitated the design and analysis of the experiments. All authors contributed to the article and approved the submitted version.

## Funding

This study was funded by the National Natural Science Foundation of China (81471197 and 81070950).

## Conflict of interest

The authors declare that the research was conducted in the absence of any commercial or financial relationships that could be construed as a potential conflict of interest.

## Publisher's note

All claims expressed in this article are solely those of the authors and do not necessarily represent those of their affiliated organizations, or those of the publisher, the editors and the reviewers. Any product that may be evaluated in this article, or claim that may be made by its manufacturer, is not guaranteed or endorsed by the publisher.
